# Epithelial-mesenchymal transition is the main way in which glioma-associated microglia/macrophages promote glioma progression

**DOI:** 10.3389/fimmu.2023.1097880

**Published:** 2023-03-10

**Authors:** Xin He, Yuduo Guo, Chunjiang Yu, Hongwei Zhang, Shengdian Wang

**Affiliations:** ^1^ Department of Neurosurgery, The Third Medical Centre, Chinese People’s Liberation Army (PLA) General Hospital, Beijing, China; ^2^ Chinese Academy of Sciences (CAS) Key Laboratory of Infection and Immunity, Institute of Biophysics, Chinese Academy of Sciences, Beijing, China; ^3^ Department of Neurosurgery, Sanbo Brain Hospital, Capital Medical University, Beijing, China

**Keywords:** glioma, glioma-associated microglia/macrophages, epithelial-mesenchymal transition, tumor microenvironment, glioma progression

## Abstract

Microglia/macrophages make up the largest population of tumor-infiltrating cells. Numerous studies have demonstrated that glioma-associated microglia/macrophages (GAMs) could promote the malignant progression of gliomas in various pathways. However, the primary function of GAMs in glioma remains inconclusive. First, by the CIBERSORT algorithm, we evaluated the content of microglia/macrophages in glioma tissues by bioinformatic analysis of omic data from thousands of glioma samples. Subsequently, we analyzed and confirmed the significant relationship between GAMs and the malignant phenotype of glioma, including survival time, IDH mutation status, and time of symptom onset. Afterward, Epithelial-Mesenchymal Transition (EMT) was identified by Gene Set Enrichment Analysis (GSEA) from numerous biological processes as the most relevant mechanism of malignant progression to GAMs. Moreover, a series of clinical samples were detected, including normal brain and various-grade glioma tissues. The results not only showed that GAMs were significantly associated with gliomas and their malignancy but also that GAMs were highly correlated with the degree of EMT in gliomas. In addition, we isolated GAMs from glioma samples and constructed co-culture models (*in vitro*) to demonstrate the promotion of the EMT process in glioma cells by GAMs. In conclusion, our study clarified that GAMs exert oncogenic effects with EMT in gliomas, suggesting the possibility of GAMs as immunotherapeutic targets.

## 1 Introduction

Glioma is the most common malignant brain tumor and accounts for about 70% of the central nervous system’s tumors. Although applying microsurgery, radiotherapy, and chemotherapy for glioma therapy is extensive, the prognosis remains very poor, especially for high-grade glioma, with a five-year survival of less than 10% (1, 2). Recent studies have demonstrated that a large number of non-tumor cells in the tumor bulk, including cancer-associated fibroblasts (CAFs), tumor-infiltrating lymphocytes (TILs), etc., serve a crucial role in the malignant progression of tumors (3). Glioma has been characterized as a “cold” tumor containing almost no tumor-killing active immune cells, such as CD8+ T cells and Th1 cells (4, 5). Instead, the most infiltrating immune cells in gliomas are myeloid cells, including macrophages and microglia, contributing to one-third of the total tumor mass (6–8). Moreover, glioma-associated microglia/macrophages (GAMs), as tumor-supportive cells, have been shown to promote glioma progression in various ways (6, 9, 10). For example, one study proposed a CCL2/CCR2/interleukin-6 axis between glioma cells and GAMs, which could promote tumor invasiveness (11). Previous studies have reported that GAMs could release factors, such as epidermal growth factor (EGF), and transforming growth factor-β (TGF-β), to increase the migration and growth of glioma cells (9, 12). However, the most prominent functional regulation of glioma cells by GAMs remains to be elucidated.

In our study, we clarified the relationship between GAMs and the malignancy of gliomas. Subsequently, the bioinformatic analysis demonstrated that the epithelial-mesenchymal transition (EMT), a critical morphological event where polarized epithelial cells convert to contractile and motile mesenchymal cells, was most associated with the infiltration of GAMs (13). Furthermore, the close relationship between GAMs and EMT was verified by clinical samples, and the effect of GAMs on the EMT process of glioma cells was confirmed by *in vitro* experiments. In conclusion, our study reveals that EMT is the predominant mechanism by which GAMs regulate the malignant progression of glioma cells, among numerous complex regulatory mechanisms, and provides valuable theoretical support for glioma immunotherapy targeting GAMs.

## 2 Results

### 2.1 The infiltration of GAMs was significantly associated with the malignancy of glioma

**Figure 1:**
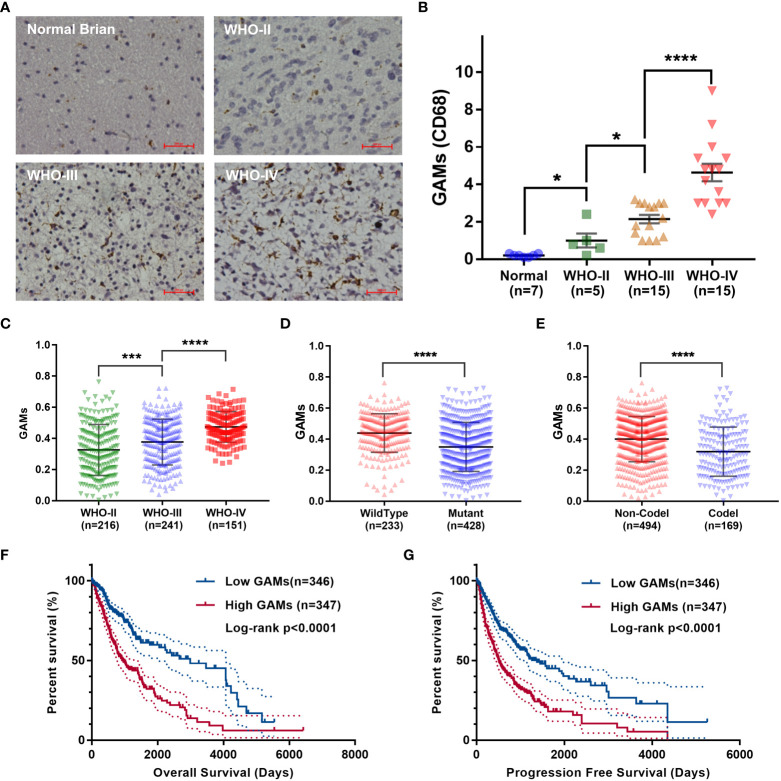
Analysis of correlation between the infiltration of GAMs and glioma malignancy. **(A, B)**. Detection of CD68 expression in normal brain tissues and glioma tissues with different malignant grades. The representative IHC of normal and glioma tissues **(A)**. The statistical graphs of GAMs (CD68^+^) **(B)**. **(C-G)**. Analysis of RNA-seq of glioma samples from the TCGA dataset. The levels of GAMs in gliomas with different WHO grades **(C)**, IDH mutation **(D)**, and 1p19q deletion **(E)** status. Correlation between the contents of GAMs and the overall survival **(F)** and progression-free survival **(G)** of glioma patients. (Scale bars represent 200 μm; * for p-value< 0.05; *** for p-value< 0.01; **** for p-value< 0.0001).

To clarify the role of GAMs in gliomas, we performed immunohistochemistry (IHC) to detect the infiltration of GAMs in samples, including 35 gliomas and seven normal brain tissues. As shown in **Figures 1A, B**, the content of CD68-labeled GAMs in glioma samples was higher than that in normal brain tissue and positively correlated with glioma grade. Moreover, we found that the progression of glioma with high GAMs was more rapid compared to patients with low levels of GAMs, as evidenced by more severe edema (7.11 ± 1.58 vs. 5.59 ± 2.12) and earlier onset of symptoms (5.44 ± 4.86 days vs. 12.14 ± 3.13 days) (**Table S1**).

We also analyzed RNA-seq or RNA-array data of thousands of glioma samples from several datasets for the correlation between the contents of GAMs and the malignancy of glioma. Consistent with the results of our clinical samples, the number of GAMs increased with the higher grade of glioma malignancy (**Figures 1C**, **S1A, F, I**). It is well known that wild-type isocitrate dehydrogenase 1 and 2 (IDH1 and IDH2, hereafter collectively referred to as IDH) and 1p19q non-deletion are two important genetic events in gliomas closely associated with rapid progression and high recurrence (14). Therefore, we examined the association of GAMs with these pathological features of gliomas. As shown in **Figures 1D, E**, the level of GAMs in gliomas with wild-type IDH or without 1p19q codeletion was higher than that of gliomas with mutant IDH or with codeletion of genome 1p-19q (**Figures S1D, E, H**). Meanwhile, our analysis showed that gliomas with higher GAMs infiltration had a worse prognosis in terms of overall survival and progression-free survival (**Figures 1F, G**, **S1B, G, J**). The recurrent gliomas also had higher GAMs infiltration than primary gliomas (**Figure S1C**). All these results suggested the critical role of GAMs in the malignant progression of gliomas.

### 2.2 Identification of the biological effects of GAMs on glioma malignancy

**Figure 2:**
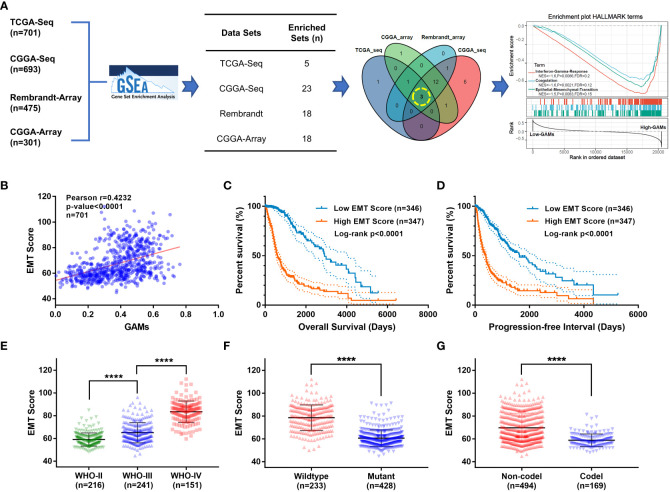
Investigation of the most prominent functions of GAMs in glioma samples *via* bioinformatics analysis. **(A)** Flow diagram of the bioinformatics investigation of biological functions associated with GAMs in gliomas: GSEA was performed on each glioma dataset (TCGA-seq, CGGA-seq, CGGA-array, and Rembrandt-array) to screen for functional gene sets associated with infiltration of GAMs, then the Venn diagram selected the gene sets from GSEA results of multiple datasets. (A. right) The enrichment plot of the three gene sets in samples from the TCGA-seq dataset ranked by GAMs. **(B-G)** Validation of the clinical features of EMT in the TCGA-seq dataset. **(B)** Correlation between EMT scores and GAMs content of glioma samples. The overall survival **(C)** and progression-free survival **(D)** plots for the glioma patients with different EMT scores. The EMT scores of gliomas with different characteristics, including WHO grades **(E)**, IDH mutation **(F)**, and 1p19q co-deletion **(G)** status. (**** for p-value< 0.0001).

To explore the potential biological effects of GAMs on glioma malignancy, the CIBERSORT algorithm (15) was used to measure the amount of GAMs in samples from four independent glioma datasets, and then the Gene Set Enrichment Analysis (GSEA) was performed for the levels of GAMs in the samples. The gene sets that are potentially related to the levels of GAMs in glioma samples were obtained from each dataset (TCGA-Seq: 5, CGGA-Seq: 23, Rembrandt-Array: 18, CGGA-Array: 18). To identify the most core biological functions to GAMs, taking intersection was performed from the obtained gene sets to select the common parts. As shown in the Venn diagram (**Figure 2A**), three gene sets were finally filtered, including “Interferon-Gamma-Response”, “Coagulation”, and “Epithelial-Mesenchymal-Translation” (EMT). Moreover, these three functional gene sets were significantly enriched in gliomas (from the TCGA dataset) with high GAMs (**Figure 2A**, right). Because EMT is an essential process in tumor cell motility and migration that promotes tumor invasion, which is one of the most critical malignant phenotypes of glioma (16, 17), we further scored each of the glioma profiles for “EMT-ness” in multiple datasets by Creighton’s signature (18). The EMT scores showed a significant positive correlation with GAMs infiltration (**Figures 2B**, **S2A**). Moreover, the patients with high EMT scores suffered a worse survival than those with low EMT scores (**Figures 2C, D**, **S2B**). In addition, the EMT score was more prominent in the more malignant subtypes of glioma, including high grade, non-codeletion of 1p-19q, or IDH wild-type gliomas (**Figures 2E–G**, **S2C, D**). These results suggested that the infiltration level of GAMs in gliomas was significantly correlated with the EMT degree, which affected the malignant progression and the prognosis of gliomas.

### 2.3 Validation of the relationship between GAMs and EMT in clinical samples

**Figure 3:**
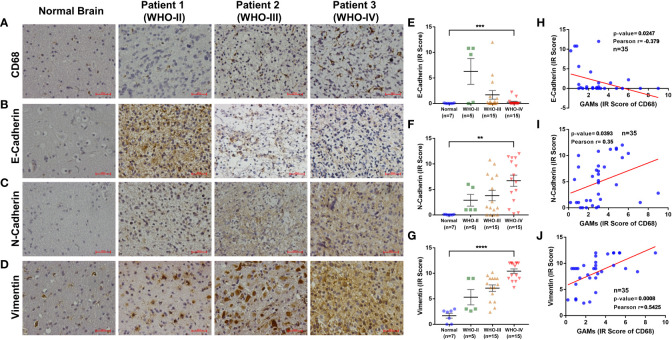
Analysis of the relationship between the degree of EMT and infiltration of GAMs in glioma samples. **(A-G)** Detection of the content of GAMs and the degree of EMT in normal brain and different grade glioma tissues by IHC. Representative IHC of GAMs (CD68^+^) in normal brain and different grade glioma tissues **(A)**. Representative IHC and statistical graphs of expression of EMT markers, E-cadherin **(B, E)**, N-cadherin **(C, F)**, and Vimentin **(D, G)** in normal brain and different grade glioma tissues. **(H-J)** The relationships between GAMs and EMT markers’ expression in glioma tissues, including E-cadherin **(H)**, N-cadherin **(I)**, and Vimentin **(J)**. (Scale bars represent 200 μm; ** for p-value<0.01, *** for p-value< 0.001, and **** for p-value< 0.0001).

To confirm the underlying relationship between GAMs and EMT processes in gliomas, we collected an independent sample set and examined the content of GAMs and the expression of EMT markers by IHC. As the degree of malignancy (WHO grade) increased, not only did the content of GAMs increase (**Figure 3A**), but the EMT markers exhibited significant changes, including the decrease of E-cadherin and the increase of N-cadherin and Vimentin (**Figures 3B–G**). Notably, as shown in **Figures 3H–J**, there were significant correlations between the content of GAMs and the expression of EMT markers. The expression of E-cadherin was negatively related to the level of GAMs infiltration (**Figure 3H**). On the contrary, the expression of N-cadherin and Vimentin were positively correlated with the infiltration of GAMs (**Figures 3I, J**). Interestingly, the correlation remained valid even in different regions in the same sample (**Figure S3**). These results indicated a critical role for GAMs in the EMT process of glioma.

### 2.4 GAMs could promote the invasion of glioma cells by regulating the EMT process

**Figure 4:**
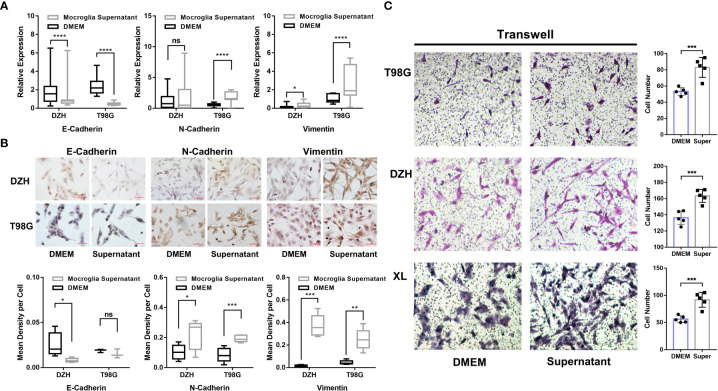
Investigation of GAMs’ effect on the EMT process and invasion of glioma cells. **(A, B)** The glioma cell lines (DZH and T98G) were cultured with the supernatants of GAMs or DMEM media as a negative control for 12 hours. The expression of EMT markers in glioma cells was detected by q-PCR and immunocytochemistry (ICC). **(A)** The expression levels of E-Cadherin, N-Cadherin, and Vimentin were detected by q-PCR. **(B)** Representative ICC detection of E-Cadherin, N-Cadherin, and Vimentin in glioma cells, and the corresponding statistical graphs were shown in the bottom row. **(C)** The effect of GAMs’ supernatant on the invasion capacity of three glioma cell lines (T98G, DZH, and XL) was investigated by transwell assay (DMEM as control). The representative transwell images of different glioma cell lines (left) and corresponding statistical graphs (right) were shown. (* for p-value<0.05, ** for p-value< 0.01, and *** for p-value< 0.001).

The above results suggested a potential correlation between GAMs and the process in gliomas. Therefore, *in vitro* experiments were performed to investigate the regulatory effects of GAMs on the EMT process of glioma cells. GAMs (CD68^+^) were isolated from glioma samples and cultured to obtain conditioned media (**Figure S4**). Then, we co-cultured glioma cells with the conditioned media to investigate the effects of soluble factors secreted by GAMs on the EMT of glioma cells. As shown in **Figures 4A, B**, conditioned media from GAMs significantly upregulated N-Cadherin and Vimentin, whereas downregulated E-Cadherin’s expression of RNA and protein in glioma cells. Moreover, the supernatant of GAMs significantly promoted the migration of glioma cells (**Figure 4C**). These results demonstrated that infiltration of macrophages could increase the malignant progression of gliomas by promoting the EMT process of glioma cells.

## 3 Discussion

Glioma is the most common and lethal brain tumor in humans with poor survival. Advances in conventional therapies, including surgery, radiotherapy, and chemotherapy, have had minimal impact on the prognosis of this aggressive disease (1). Studies have demonstrated the pivotal role of infiltrating immune cells in the malignant progression of gliomas, while GAMs are the predominant infiltrating immune cells in malignant glioma and account for up to 40% of the tumor mass (6–8). And GAM density was increased in high-grade tumors that correlated with a pro-tumorigenic molecular signature (19). GAMs are believed to originate from two distinct sources. Among them is resident microglia, a particular lineage arising from embryonic yolk sac myelomonocytes (20). The second group of immune cell macrophage precursors in the CNS are peripheral bone marrow-derived mononuclear cells. Accumulating data from numerous studies demonstrated that Tumor-associated macrophages (TAMs) represent one of the main tumor-infiltrating immune cell types and are generally categorized into either of two functionally contrasting subtypes, namely classical activated M1 macrophages and alternatively activated M2 macrophages (21, 22). Human GBMs contain mixed M1/M2-like polarized GAMs. Previous studies demonstrated that TAMs are predominantly M2-like macrophages associated with cancer progression (23, 24). However, may be due to its extreme plasticity, recent studies showed that M2/M1-like macrophages associated with survival and outcomes are currently questioned in other types of tumors (25, 26). GAMs are believed to be essential in creating a local tumor microenvironment that is immunosuppressive and promotes glioma progression (27). For example, GAMs could exert immunomodulation effects by secreting potent immunosuppressive cytokines IL-10, IL-6, and TGF-beta. And GAMs could induce programmed death and suppress the immune response of lymphocytes by expressing FasL and B7-H1 (28–30). GAMs also induce a transition of glioblastoma cells into mesenchymal-like (MES-like) States (31). However, the relationship between GAMs and the malignancy of gliomas, and the most predominant way in which GAMs promote the progression of gliomas remain to be elucidated.

In the present study, we examined the GAMs infiltration in different WHO grades by IHC. We confirmed that GAMs infiltration levels were higher in 35 glioma samples than in 7 normal brain tissues. We also observed that the level of GAMs infiltration was positively correlated with tumor grading as there was a significant difference between high- and low-grade gliomas. In addition, by analyzing thousands of glioma samples, our study demonstrated the clinical significance of GAMs in glioma. The level of GAMs in gliomas has a definite correlation with molecular subtypes (IDH-mutation and 1p-19q codeletion status), as well as the prognosis of the patients (OS and PFS). Clinicopathologic characteristics analyses showed that increased infiltration of GAMs was positively associated with brain edema and shortened the time between first symptom and first examination, suggesting the rapid progression of the disease. These data indicated a vital role for GAMs in glioma progression as well as the prognosis of the patients, consistent with previous basic studies. For example, a study reported that GAMs could promote the growth of glioma cells by secreting CECR1 (32). Studies also reported that the localization of GAMs inside the vital tumor core seemed to be critical for the statistical evaluation of survival analysis (33), as well as peritumoral GAM have a unique gene signature and may influence disease outcomes by recruiting systemic monocytes to the glioma microenvironment (34). A recent study revealed interactivity between GAMs and astrocytes, which stimulated the JAK/STAT pathway in astrocytes to express various anti-inflammatory cytokines, including TGF-β, IL-10, and G-CSF, contributing to the migration and proliferation of gliomas (35). Furthermore, by analyzing and integrating the biological functions associated with GAMs in multiple independent datasets of glioma, the EMT process was suggested to be the most dominant carcinogenic role of GAMs in glioma. And our results from clinical samples showed that the degree of EMT was significantly correlated with the content of GAMs in gliomas. Moreover, in high-grade glioma consecutive sections, the area with GAMs nearby expressed significantly less E-cadherin and more N-cadherin and Vimentin and vice versa. These data indicated that the EMT process is critical for glioma progression, and GAMs may play an important role. To investigate the mechanism of EMT regulation by GAMs, we established two glioma cell lines from two glioblastoma patients (XL and DZH). Glioma cell line XL strongly expressed the E-cadherin. Together with the classical GBM cell line T98G, these two glioma cell lines were used to analyze the invasion and EMT affection by GAMs supernatant. Our results showed that the GAMs supernatant significantly increased the tumor invasion in all three glioma cell lines. We also observed that the EMT marks expression was fully and partially changed in these three cell lines in the RNA and protein levels.

In conclusion, this study provides evidence that GAMs is an effective factor for regulating glioma cell invasion by promoting the EMT process and presents GAMs as an tool for prognostic evaluation. However, our current study has some limitations. The biggest limitation of the current study is that we did not identify the GAMs subgroups which may potential associated with overall survival of patients (33). And the mechanism of GAMs promoting glioma EMT has not been studied in depth. Therefore, further experiments are needed to assess the underlying mechanism of our findings in this study.

## 4 Materials and methods

### 4.1 Bioinformatics analysis

All transcriptome datasets in this study, including RNA-seq (TCGA-Seq, CGGA-Seq) and Array (CGGA-Array, Rembrandt-Array), and corresponding clinical information were obtained from The Cancer Genome Atlas (TCGA, https://portal.gdc.cancer.gov/), Chinese Glioma Genome Atlas (CGGA, http://www.cgga.org.cn/), and Gene-Expression Omnibus (GEO-GSE108474, https://www.ncbi.nlm.nih.gov/geo/). These data were preprocessed by R software (v5.1) with the Limma package, including data washing and normalization (36). The content of GAMs in the tumors was assessed by the CIBERSORT algorithm (15). The “EMT score” of samples was calculated by the method reported by Chad J. Creighton (18). Gene Set Enrichment Analysis (GSEA) was performed by GSEA software (v3.1), and hallmark gene sets were used as reference (c1.all.v7.0.symbols.gmt) (37). |NES|>1, False discovery rate (FDR)< 0.25 and p-value< 0.05 were considered significantly enrichment.

### 4.2 Human glioma tissues and cell lines

This study was approved by the Ethics Committee of Sanbo Brain Hospital, Capital Medical University (SBNK-YJ-2020-001-01). Primary glioma tissues were collected from 35 patients at the Sanbo Brain Hospital, Capital Medical University. Diagnosis of gliomas was established histologically according to the 2016 WHO classification. Seven normal brain tissues were obtained from the patients undergoing emergency cranial surgery, where some of the brain tissue needed to be removed to ensure that the surgery could be performed properly to save lives. According to the preoperative MRI (enhanced T1WI, T2WI) of the patients, the maximum diameter and vertical diameter of the tumor in axial position (marked as a and b), as well as the maximum height in coronal position (marked as c), were measured respectively. According to the formula: $V=\frac{4}{3}\pi \times abc$ , the size of the lesion (V edema + V tumor) by T2WI, and the size of the tumor itself (enhanced T1WI, V tumor) were calculated, respectively. The edema index (EI) was calculated according to the formula: $EI=\frac{V\left(edema\right)+V\left(tumor\right)}{V\left(tumor\right)}$ (38). Human glioma cell line T98G was obtained from the Chinese Academy of Sciences cell bank. In this study, we established two glioma cell lines from two glioblastoma patients named XL and DZH. Primary GBM tumor cell lines XL and DZH (uniformly expressing the glial fibrillary acidic protein, GFAP^+^) were obtained by culture and expansion of *ex vivo* tumor specimens in the cell culture medium. Cells were used after 10–15 passages. All the glioma cell lines were cultured in Dulbecco’s modified Eagle’s medium (DMEM) supplemented with 10% fetal bovine serum (FBS), 100 units/ml penicillin, and 100 ng/ml streptomycin. All cells were incubated at 37°C in an atmosphere of 5% CO_2_.

### 4.3 Immunohistochemistry

Paraffin sections (4 μm) from samples were deparaffinized in 100% xylene and re-hydrated in descending ethanol series and water according to standard protocols. Heat-induced antigen retrieval was performed in 10 mM citrate buffer for 2 min at 100°C. Endogenous peroxidase activity and non-specific antigens were blocked with peroxidase blocking reagent containing 3% hydrogen peroxide and serum, followed by incubation with rabbit anti-human CD68 antibody (1: 200) (Abcam, United Kingdom), rabbit anti-human E-cadherin antibody (1: 150) (Zhongshanjinqiao, China), rabbit anti-human N-cadherin antibody (1: 150) (Zhongshanjinqiao, China), rabbit anti-human Vimentin antibody (1: 150) (Zhongshanjinqiao, China), overnight at 4°C. After washing, the sections were incubated with the biotin-labeled rabbit anti-goat antibody for 30 min at 37°C. The peroxidase reaction was developed using 3,3-diaminobenzidine (DAB) chromogen solution in the DAB buffer substrate. Sections were visualized with DAB, counterstained with hematoxylin, mounted in neutral gum, and analyzed using a bright field microscope.

### 4.4 Isolation, culture, and identification of GAMs from glioma tissue

Primary GAMs were prepared from high-grade fresh glioma tissue (WHO III-IV) as previously described (39–41). In brief, cells were isolated from gliomas by density-gradient centrifugation followed by immunomagnetic beads with CD11b and were suspended in the conditioned culture medium and plated at a final density of 5×10^5^ cells/cm^2^. GAMs supernatant was harvested after 24 h for further studies. Conditioned media were prepared as follows: 24 h after seeding 1×10^6^ XL cells or onto 100-mm dishes, the standard culture medium was exchanged for 8 mL of high-glucose DMEM with 10% FBS and GlutaMax (a medium used for a GAMs culture). Conditioned media were harvested after 24 h from 85 to 90% confluent cultures and centrifuged at 300g for 10 min to remove cell debris. Freshly prepared conditioned mediums were added to GAMs cultures.

### 4.5 Cell migration assays

For the cell migration assay, 1×10^4^ cells in 100 μL DMEM medium without FCS were seeded on a fibronectin-coated polycarbonate membrane insert in a Transwell apparatus (Costar, MA). In the lower chamber, 600 μL DMEM with 10% FBS was added as a chemoattractant. After the cells were incubated for 12 h at 37°C in a 5% CO_2_ atmosphere, the insert was washed with PBS, and cells on the top surface of the insert were removed with a cotton swab. Cells adhering to the lower surface were fixed with methanol, stained with crystal violet, and counted under a microscope in five predetermined fields (200×). All assays were independently repeated at least three times.

### 4.6 Real-time quantitative PCR (RT-qPCR)

Total RNA was extracted from the cells with the TRIZOL reagent (Invitrogen, Camarillo, CA, USA). The total RNA (1 mg) from each sample was reverse-transcribed into cDNA with the M-MLV reverse transcriptase (Promega, Madison, WI, USA) according to the manufacturer’s instructions. Real-time qPCR was performed with SYBR Green (TransGen Biotech, Osaka, Japan) on an ABI 7500 Real-Time PCR System (Applied Biosystems, Foster City, CA, USA) according to the manufacturer’s protocol. The primers were designed using Primer Premier 5.0 software (PREMIER Biosoft International, Palo Alto, CA, USA) and are listed in **Table 1**. The amplification was conducted under the following conditions: 95°C for 30s, 95°C for 5s, 55°C for 10s, and 72°Cfor 45s for 35 cycles. In each PCR reaction, nuclease-free water (TIANGEN, Beijing, China) was substituted for the cDNA as a negative control. The relative gene expression was determined using the 2^-ΔΔCt^ method according to the manufacturer’s recommended protocol.

**Table 1:** The primer sequence of EMT marks.

Primer		Sequence (5’ to 3’)
**E-Cadherin**	Forward	GCCTCCTGAAAAGAGAGTGGAAG
	Reverse	TGGCAGTGTCTCTCCAAATCCG
**N-Cadherin**	Forward	CCTCCAGAGTTTACTGCCATGAC
	Reverse	GTAGGATCTCCGCCACTGATTC
**Vimentin**	Forward	AGGCAAAGCAGGAGTCCACTGA
	Reverse	ATCTGGCGTTCCAGGGACTCAT

### 4.7 Immunocytochemistry

E-cadherin, N-Cadherin, and vimentin expression were investigated with immunocytochemistry. Cells grown on glass coverslips were fixed in 3% H2O2 methyl alcohol for 5 min. After three washes in PBS, 0.2% Triton-X 100 was added to the cells. Cells were blocked with 10% goat serum and then double-stained with rabbit anti-human E-cadherin antibody (1: 150) (Zhongshanjinqiao, China), rabbit anti-human N-cadherin antibody (1: 150) (Zhongshanjinqiao, China), rabbit anti-human Vimentin antibody (1: 150) (Zhongshanjinqiao, China), overnight at 4°C. After washing, the coverslips were incubated with a biotin-labeled rabbit anti-goat antibody for 30 min at 37°C. The peroxidase reaction was developed using 3,3-diaminobenzidine (DAB) chromogen solution in the DAB buffer substrate. Finally, all cell nuclei were stained with hematoxylin. Coverslips were then mounted, and the slides were examined with a microscope

### 4.8 Evaluation of staining

The immunohistochemically stained tissue sections were reviewed and scored separately by two pathologists blinded to the clinical parameters. For immunohistochemically, the expression of CD68, E-cadherin, N-cadherin, and Vimentin were scored semi-quantitatively based on staining intensity and distribution using the immunoreactive score (IRS). Briefly, Immunoreactive score (IRS) = SI (staining intensity) PP (percentage of positive cells).SI was assigned as: 0= negative; 1= weak; 2= moderate; 3= strong. PP is defined as 0 = 0%; 1 = 0-25%; 2 = 25-50%; 3 = 50-75%; 4 = 75-100%. For categorization of the continuous CD68 values into low and high, we chose a commonly used cutoff point for the measurements (range 0–12, cut point ≤ 3 versus > 3). For immunocytochemistry, the IOD (integrated optical density) and whole-cell area in each view field (area sum) were analyzed by IPP (Image-Pro Plus) software. The expression of CD68, E-cadherin, N-cadherin, and Vimentin were scored semi-quantitatively based on average IOD (average IOD= IOD/area sum). Both in immunohistochemistry and immunocytochemistry, Cells stained with indicated antibodies were calculated per field of view, with at least 5 view fields per section evaluated at 400× magnification.

### 4.9 Statistical analysis

All quantified data represented an average of at least triplicate samples. SPSS 13.0 and Graph Pad Prism 5.0 were used for statistical analysis. Data are presented as mean ± SD. One-way ANOVA or two-tailed Student’s t-test was used for comparisons between groups. Pearson correlation analysis was used to evaluate the linear correlation between the two variables. Kaplan-Meier curve and Log-rank test were applied for survival analysis. Differences were considered statistically significant when P< 0.05.

## Data availability statement

The original contributions presented in the study are included in the article/**Supplementary Material**. Further inquiries can be directed to the corresponding authors.

## Ethics statement

The studies involving human participants were reviewed and approved by Ethics Committee of Sanbo Brain Hospital of Capital Medical University. The patients/participants provided their written informed consent to participate in this study. Written informed consent was obtained from the individual(s) for the publication of any potentially identifiable images or data included in this article.

## Author contributions

XH collected clinical samples and completed basic experiments, YG performed the data analysis and wrote the manuscript. CY provided support for clinical medical knowledge. HZ and SW designed and supervised the study. All authors contributed to the article and approved the submitted version.

## Supplementary material

Supplementary Figure 1 (Correspondence with **Figure 1**) Analysis of the relationship between GAMs and clinical features of gliomas from multiple datasets. (CGGA-Seq, CGGA-Array, and Rembrandt-Array). The levels of GAMs in gliomas with different characteristics, including WHO-grade **(A, F, I)**, IDH mutation **(D, H)**, 1p19q-codeletion **(E)**, and primary or recurrent **(C)**. Survival plots of glioma patients with different GAMs levels **(B, G, J)**.

Supplementary Figure 2 (Correspondence with **Figure 2**) Analysis of the relationship between EMT and malignancy of gliomas from CGGA-array dataset. **(A)** Correlation between GAMs and EMT scores of glioma samples. **(B)** The survival curves of patients with different EMT scores. The EMT scores of gliomas with different WHO grades **(C)** and IDH mutation status **(D)**.

Supplementary Figure 3 (Correspondence with **Figure 3**) The expression of EMT markers in GAMs in glioma samples. Representative IHC staining of CD68, E-Cadherin, N-Cadherin, and Vimentin in consecutive sections of a glioma tissue (WHO-III). (Scale bars represent 500 μm).

Supplementary Figure 4 (Correspondence with **Figure 4**) The characterization of GAMs isolated from the glioma tissue. **(A)** The CD45+CD11b+ cells (GAMs) were sorted from single cell suspension of glioma tissues. **(B, C)** Representative ICC of sorted cells stained with CD68 **(B)**, or without primary antibody **(C)**.

## References

[1] Ohgaki H Kleihues P. Epidemiology and etiology of gliomas. Acta Neuropathol (2005) 109:93–108. doi: 10.1007/s00401-005-0991-y 15685439

[2] Larson EW. Clinical outcomes following salvage gamma knife radiosurgery for recurrent glioblastoma. World J Clin Oncol (2014) 5:142. doi: 10.5306/wjco.v5.i2.142 24829861 PMC4014786

[3] Turley SJ Cremasco V Astarita JL. Immunological hallmarks of stromal cells in the tumour microenvironment. Nat Rev Immunol (2015) 15:669–82. doi: 10.1038/nri3902 26471778

[4] Gajewski TF Schreiber H Fu Y-X. Innate and adaptive immune cells in the tumor microenvironment. Nat Immunol (2013) 14:1014–22. doi: 10.1038/ni.2703 PMC4118725 24048123

[5] Keskin DB Anandappa AJ Sun J Tirosh I Mathewson ND Li S. Neoantigen vaccine generates intratumoral T cell responses in phase ib glioblastoma trial. Nature (2019) 565:234–9. doi: 10.1038/s41586-018-0792-9 PMC6546179 30568305

[6] Hambardzumyan D Gutmann DH Kettenmann H. The role of microglia and macrophages in glioma maintenance and progression. Nat Neurosci (2016) 19:20–7. doi: 10.1038/nn.4185 PMC4876023 26713745

[7] Kennedy BC Maier LM D’Amico R Mandigo CE Fontana EJ Waziri A. Dynamics of central and peripheral immunomodulation in a murine glioma model. BMC Immunol (2009) 10:11. doi: 10.1186/1471-2172-10-11 19226468 PMC2654428

[8] Kostianovsky AM Maier LM Anderson RC Bruce JN Anderson DE. Astrocytic regulation of human Monocytic/Microglial activation. J Immunol (2008) 181:5425–32. doi: 10.4049/jimmunol.181.8.5425 18832699

[9] Coniglio SJ Eugenin E Dobrenis K Stanley ER West BL Symons MH. Microglial stimulation of glioblastoma invasion involves epidermal growth factor receptor (EGFR) and colony stimulating factor 1 receptor (CSF-1R) signaling. Mol Med (2012) 18:519–27. doi: 10.2119/molmed.2011.00217 PMC3356419 22294205

[10] Blay J White TD Hoskin DW. The extracellular fluid of solid carcinomas contains immunosuppressive concentrations of adenosine. Cancer Res (1997) 57:2602–5. 9205063

[11] Zhang J Sarkar S Cua R Zhou Y Hader W Yong VW. And microglia that promotes tumor invasiveness through the CCL2/CCR2/interleukin-6 axis. Carcinogenesis (2012) 33:312–9. doi: 10.1093/carcin/bgr289 22159219

[12] Wick W Platten M Weller M. Glioma cell invasion: Regulation of metalloproteinase activity by TGF-β. J Neurooncol (2001) 53:177–85. doi: 10.1023/A:1012209518843 11716069

[13] Arias AM. Epithelial mesenchymal interactions in cancer and development. Cell (2001) 105:425–31. doi: 10.1016/S0092-8674(01)00365-8 11371340

[14] Louis DN Perry A Reifenberger G von Deimling A Figarella-Branger D Cavenee WK. The 2016 world health organization classification of tumors of the central nervous system: a summary. Acta Neuropathol (2016) 131:803–20. doi: 10.1007/s00401-016-1545-1 27157931

[15] Newman AM Steen CB Liu CL Gentles AJ Chaudhuri AA Scherer F. Determining cell type abundance and expression from bulk tissues with digital cytometry. Nat Biotechnol (2019) 37:773–82. doi: 10.1038/s41587-019-0114-2 PMC6610714 31061481

[16] Banyard J Bielenberg DR. The role of EMT and MET in cancer dissemination. Connect Tissue Res (2015) 56:403–13. doi: 10.3109/03008207.2015.1060970 PMC4780319 26291767

[17] Paw I Carpenter RC Watabe K Debinski W Lo HW. Mechanisms regulating glioma invasion. Cancer Lett (2015) 362:1–7. doi: 10.1016/j.canlet.2015.03.015 25796440 PMC4435977

[18] Gibbons DL Creighton CJ. Pan-cancer survey of epithelial-mesenchymal transition markers across the cancer genome atlas. Dev Dyn (2018) 247:555–64. doi: 10.1002/dvdy.24485 PMC5503821 28073171

[19] Toedebusch R Grodzki AC Dickinson PJ Woolard K Vinson N Sturges B. Glioma-associated microglia/macrophages augment tumorigenicity in canine astrocytoma, a naturally occurring model of human glioma. Neuro-Oncol Adv (2021) 3:1–13. doi: 10.1093/noajnl/vdab062 PMC8193901 34131649

[20] Ginhoux F Greter M Leboeuf M Nandi S See P Gokhan S. Fate mapping analysis reveals that adult microglia derive from primitive macrophages. Sci (80- ) (2010) 330:841–5. doi: 10.1126/science.1194637 PMC3719181 20966214

[21] Liu J Geng X Hou J Wu G. New insights into M1/M2 macrophages: key modulators in cancer progression. Cancer Cell Int (2021) 21:389. doi: 10.1186/s12935-021-02089-2 34289846 PMC8296555

[22] Pan Y Yu Y Wang X Zhang T. Tumor-associated macrophages in tumor immunity. Front Immunol (2020) 11:583084. doi: 10.3389/fimmu.2020.583084 33365025 PMC7751482

[23] Yuan R Li S Geng H Wang X Guan Q Li X. Reversing the polarization of tumor-associated macrophages inhibits tumor metastasis. Int Immunopharmacol (2017) 49:30–7. doi: 10.1016/j.intimp.2017.05.014 28550732

[24] Scaldaferri D Bosi A Fabbri M Pedrini E Inforzato A Valli R. The human RNASET2 protein affects the polarization pattern of human macrophages. vitro. Immunol Lett (2018) 203:102–11. doi: 10.1016/j.imlet.2018.09.005 30218741

[25] Schnellhardt S Erber R Büttner-Herold M Rosahl M-C Ott OJ Strnad V. Accelerated partial breast irradiation: Macrophage polarisation shift classification identifies high-risk tumours in early hormone receptor-positive breast cancer. Cancers (Basel) (2020) 12:446. doi: 10.3390/cancers12020446 32075091 PMC7072550

[26] Oshi M Tokumaru Y Asaoka M Yan L Satyananda V Matsuyama R. M1 macrophage and M1/M2 ratio defined by transcriptomic signatures resemble only part of their conventional clinical characteristics in breast cancer. Sci Rep (2020) 10:16554. doi: 10.1038/s41598-020-73624-w 33024179 PMC7538579

[27] Zhai H Heppner FL Tsirka SE. Microglia/macrophages promote glioma progression. Glia (2011) 59:472–85. doi: 10.1002/glia.21117 PMC3080032 21264953

[28] Li W Graeber MB. The molecular profile of microglia under the influence of glioma. Neuro Oncol (2012) 14:958–78. doi: 10.1093/neuonc/nos116 PMC3408253 22573310

[29] Filipazzi P Huber V Rivoltini L. Phenotype, function and clinical implications of myeloid-derived suppressor cells in cancer patients. Cancer Immunol Immunother (2012) 61:255–63. doi: 10.1007/s00262-011-1161-9 PMC11029611 22120756

[30] Hussain SF Yang D Suki D Grimm E Heimberger AB. Innate immune functions of microglia isolated from human glioma patients. J Transl Med (2006) 4:15. doi: 10.1186/1479-5876-4-15 16573834 PMC1501057

[31] Hara T Chanoch-Myers R Mathewson ND Myskiw C Atta L Bussema L. Interactions between cancer cells and immune cells drive transitions to mesenchymal-like states in glioblastoma. Cancer Cell (2021) 39:779–792.e11. doi: 10.1016/j.ccell.2021.05.002 34087162 PMC8366750

[32] Zhu C Mustafa D Zheng P van der Weiden M Sacchetti A Brandt M. Activation of CECR1 in M2-like TAMs promotes paracrine stimulation-mediated glial tumor progression. Neuro Oncol (2017) 19:now251. doi: 10.1093/neuonc/now251 PMC5464467 28453746

[33] Zeiner PS Preusse C Golebiewska A Zinke J Iriondo A Muller A. Distribution and prognostic impact of microglia/macrophage subpopulations in gliomas. Brain Pathol (2019) 29:513–29. doi: 10.1111/bpa.12690 PMC6849857 30506802

[34] Caponegro MD Oh K Madeira MM Radin D Sterge N Tayyab M. A distinct microglial subset at the tumor–stroma interface of glioma. Glia (2021) 69:1767–81. doi: 10.1002/glia.23991 PMC8113099 33704822

[35] Henrik Heiland D Ravi VM Behringer SP Frenking JH Wurm J Joseph K. Tumor-associated reactive astrocytes aid the evolution of immunosuppressive environment in glioblastoma. Nat Commun (2019) 10:2541. doi: 10.1038/s41467-019-10493-6 31186414 PMC6559986

[36] Ritchie ME Phipson B Wu D Hu Y Law CW Shi W. Limma powers differential expression analyses for RNA-sequencing and microarray studies. Nucleic Acids Res (2015) 43:e47–7. doi: 10.1093/nar/gkv007 PMC4402510 25605792

[37] Subramanian A Tamayo P Mootha VK Mukherjee S Ebert BL Gillette MA. Gene set enrichment analysis: A knowledge-based approach for interpreting genome-wide expression profiles. Proc Natl Acad Sci (2005) 102:15545–50. doi: 10.1073/pnas.0506580102 PMC1239896 16199517

[38] Bitzer M Wöckel L Luft AR Wakhloo AK Petersen D Opitz H. The importance of pial blood supply to the development of peritumoral brain edema in meningiomas. J Neurosurg (1997) 87:368–73. doi: 10.3171/jns.1997.87.3.0368 9285600

[39] Agalave NM Lane BT Mody PH Szabo-Pardi TA Burton MD. Isolation, culture, and downstream characterization of primary microglia and astrocytes from adult rodent brain and spinal cord. J Neurosci Methods (2020) 340:108742. doi: 10.1016/j.jneumeth.2020.108742 32315669 PMC7293863

[40] Bohlen CJ Bennett FC Bennett ML. Isolation and culture of microglia. Curr Protoc Immunol (2019) 125:e70. doi: 10.1002/cpim.70 30414379 PMC6510657

[41] Bordt EA Block CL Petrozziello T Sadri-Vakili G Smith CJ Edlow AG. Isolation of microglia from mouse or human tissue. STAR Protoc (2020) 1:100035. doi: 10.1016/j.xpro.2020.100035 32783030 PMC7416840

