# Supplementary material for: Epithelial-mesenchymal transition is the main way in which glioma-associated microglia/macrophages promote glioma progression
Source: Front Immunol. 2023 Mar 10;14:1097880. doi: 10.3389/fimmu.2023.1097880 (PMC10036378; doi:10.3389/fimmu.2023.1097880)
Supplement: Supplementary file 1 [file Table_1.docx]

**Table S1. Correlation between the clinical characteristics and GAMs in glioma.**

| **Factor** | **Low GAMs** | **High GAMs** | **P-value** |
| --- | --- | --- | --- |
| **Edema Index** | 5.59±2.12 | 7.11±1.58 | 0.016 |
| **The time between first symptom and examination (Days)** | 12.14±3.13 | 5.44±1.86 | 0.0004 |
