# Supplementary figures and images for: Epithelial-mesenchymal transition is the main way in which glioma-associated microglia/macrophages promote glioma progression
Source: Front Immunol. 2023 Mar 10;14:1097880. doi: 10.3389/fimmu.2023.1097880 (PMC10036378; doi:10.3389/fimmu.2023.1097880)

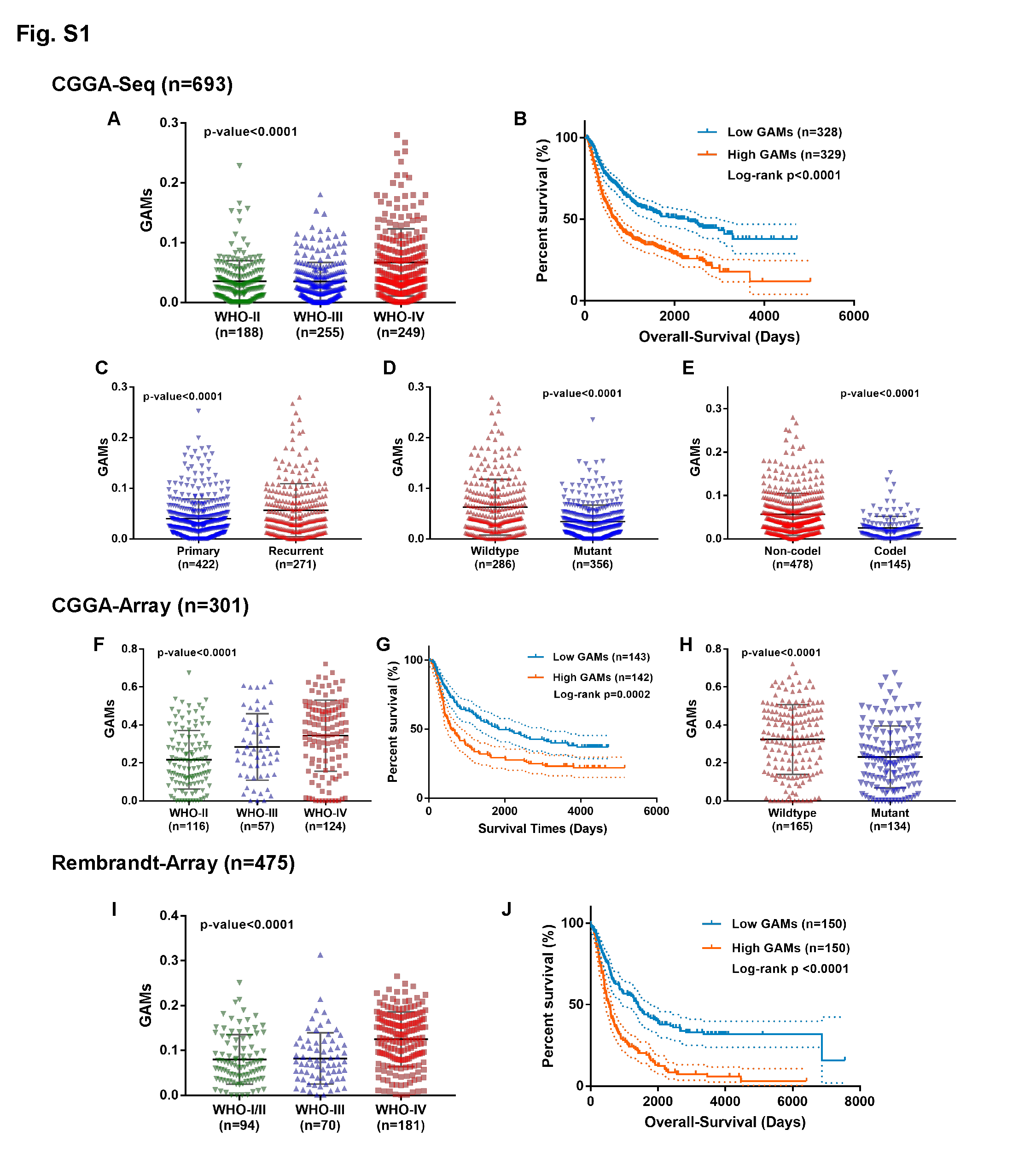

Supplement: Supplementary Figure 1 — (Correspondence with Figure 1 ) Analysis of the relationship between GAMs and clinical features of gliomas from multiple datasets. (CGGA-Seq, CGGA-Array, and Rembrandt-Array). The levels of GAMs in gliomas with different characteristics, including WHO-grade (A, F, I), IDH mutation (D, H), 1p19q-codeletion (E), and primary or recurrent (C). Survival plots of glioma patients with different GAMs levels (B, G, J). [file Image_1.tiff]

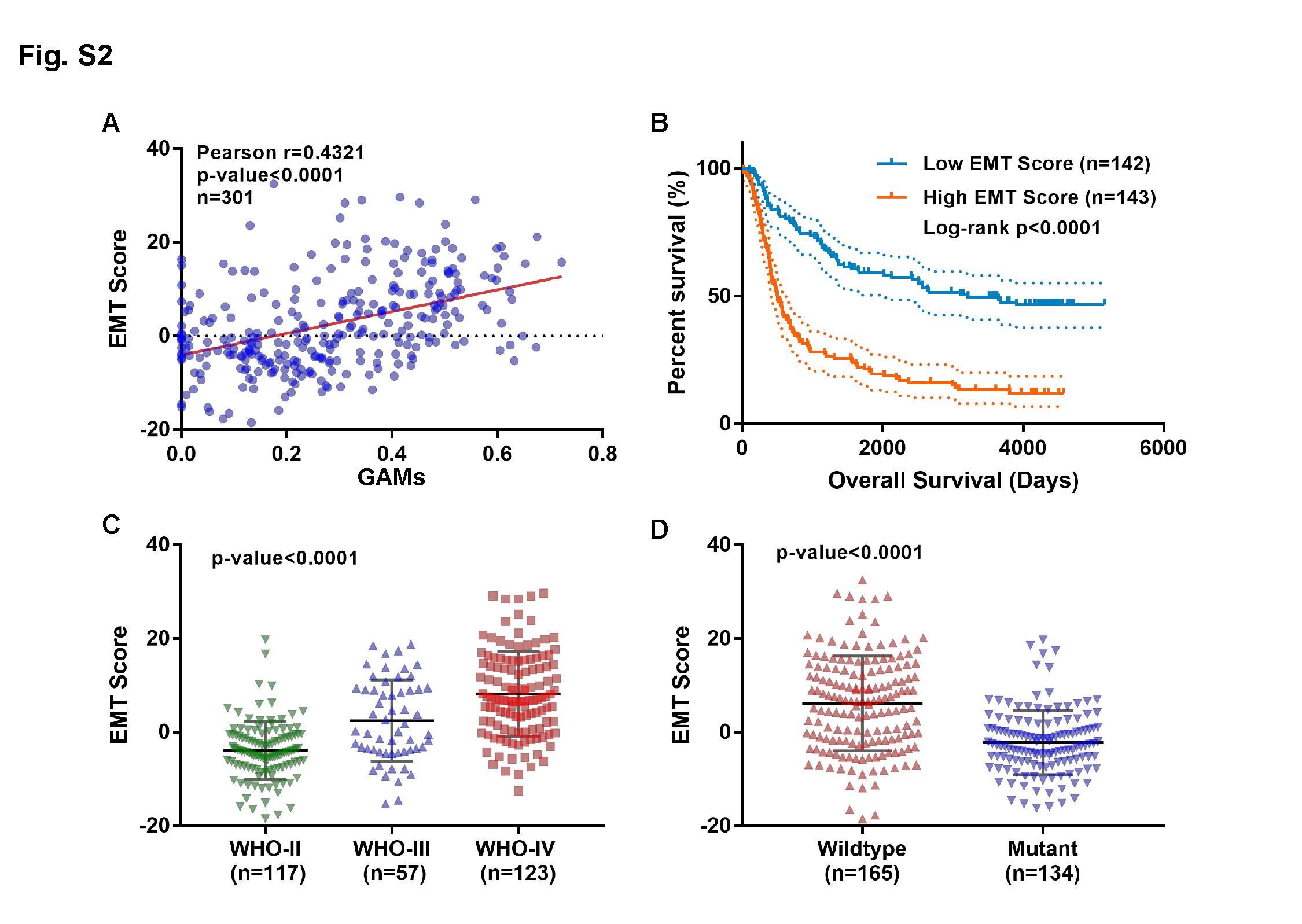

Supplement: Supplementary Figure 2 — (Correspondence with Figure 2 ) Analysis of the relationship between EMT and malignancy of gliomas from CGGA-array dataset. (A) Correlation between GAMs and EMT scores of glioma samples. (B) The survival curves of patients with different EMT scores. The EMT scores of gliomas with different WHO grades (C) and IDH mutation status (D). [file Image_2.tiff]

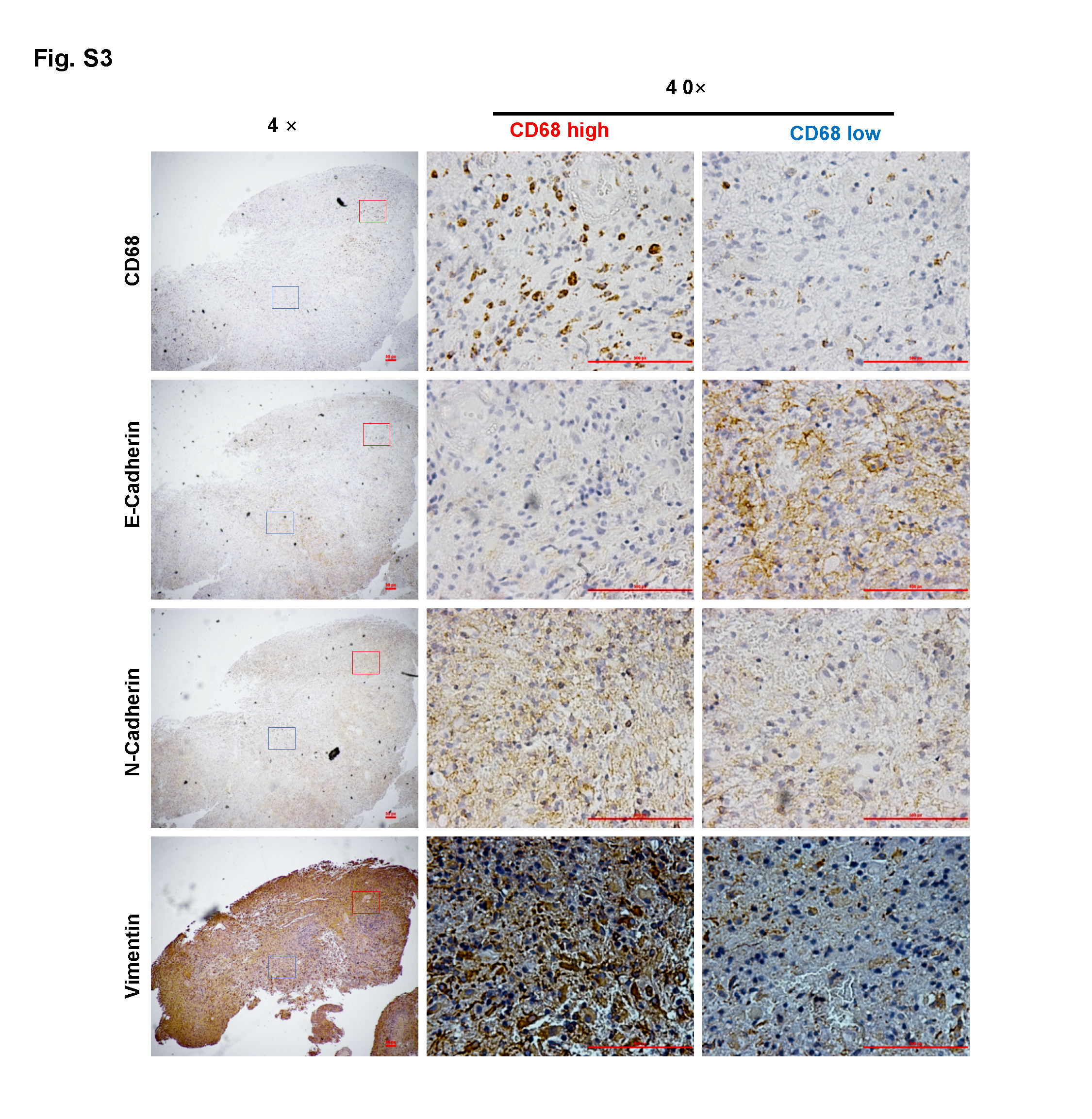

Supplement: Supplementary Figure 3 — (Correspondence with Figure 3 ) The expression of EMT markers in GAMs in glioma samples. Representative IHC staining of CD68, E-Cadherin, N-Cadherin, and Vimentin in consecutive sections of a glioma tissue (WHO-III). (Scale bars represent 500 μm). [file Image_3.tif]

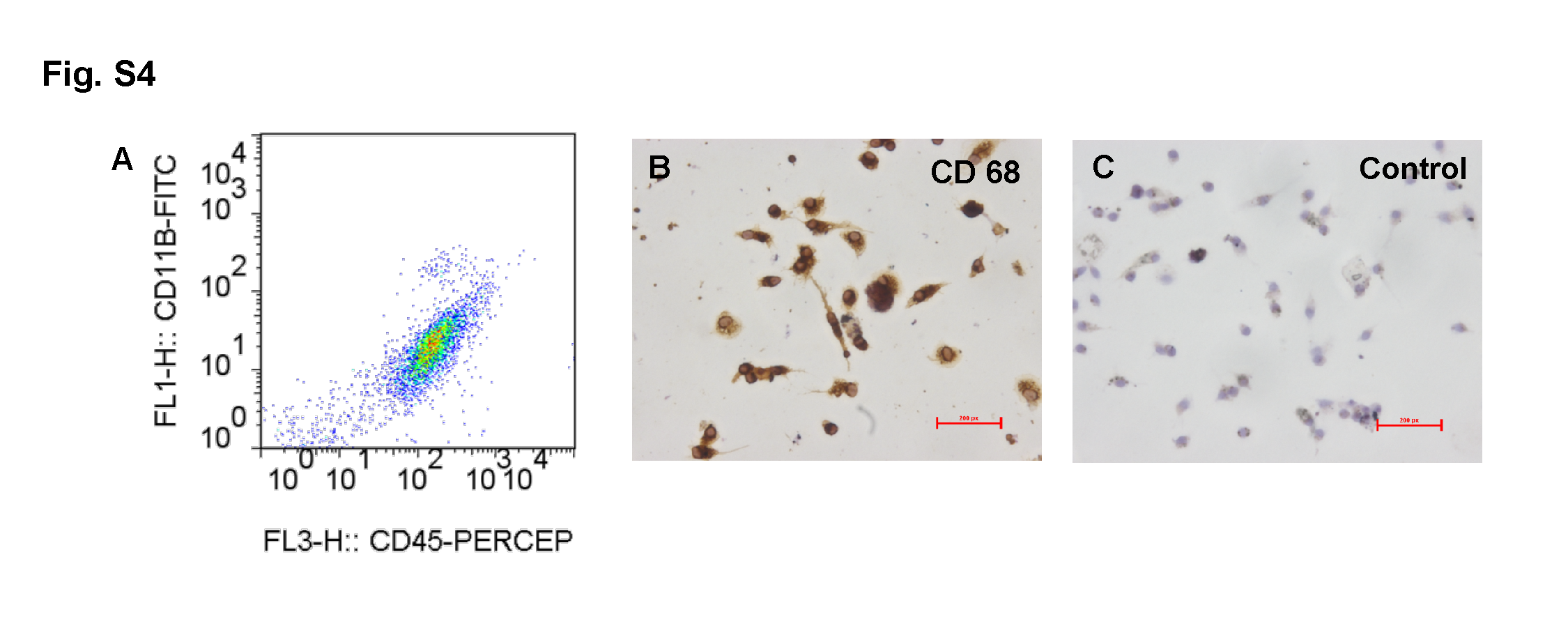

Supplement: Supplementary Figure 4 — (Correspondence with Figure 4 ) The characterization of GAMs isolated from the glioma tissue. (A) The CD45+CD11b+ cells (GAMs) were sorted from single cell suspension of glioma tissues. (B, C) Representative ICC of sorted cells stained with CD68 (B), or without primary antibody (C). [file Image_4.tiff]
